# Preclinical Evaluation of Long-Acting Emtricitabine Semi-Solid Prodrug Nanoparticle Formulations

**DOI:** 10.3390/pharmaceutics15071835

**Published:** 2023-06-27

**Authors:** Paul Curley, James J. Hobson, Neill J. Liptrott, Edward Makarov, Amer Al-khouja, Lee Tatham, Christopher A. W. David, Helen Box, Megan Neary, Joanne Sharp, Henry Pertinez, David Meyers, Charles Flexner, Caren L. Freel Meyers, Larisa Poluektova, Steve Rannard, Andrew Owen

**Affiliations:** 1 Centre of Excellence in Long-Acting Therapeutics (CELT), University of Liverpool, Liverpool L7 8TX, UK; pcurley@liverpool.ac.uk (P.C.); jayhob@liverpool.ac.uk (J.J.H.); liptrott@liverpool.ac.uk (N.J.L.); tatham@liverpool.ac.uk (L.T.); cdavid@liverpool.ac.uk (C.A.W.D.); mneary@liverpool.ac.uk (M.N.); joanne.livermore@liverpool.ac.uk (J.S.); pertinez@liverpool.ac.uk (H.P.); srannard@liverpool.ac.uk (S.R.); 2 Department of Pharmacology and Experimental Neuroscience, University of Nebraska Medical Centre, Omaha, NE 68198, USA; makarove@unmc.edu (E.M.); lpoluekt@unmc.edu (L.P.); 3 Department of Pharmacology and Molecular Sciences, Johns Hopkins School of Medicine, Baltimore, MD 21205, USA; amkhouja@gmail.com (A.A.-k.); dmeyers7@jhmi.edu (D.M.); flex@jhmi.edu (C.F.); cmeyers@jhmi.edu (C.L.F.M.)

**Keywords:** HIV, long-acting, pre-exposure prophylaxis, nano, emtricitabine

## Abstract

Long-acting injectable (LAI) formulations promise to deliver patient benefits by overcoming issues associated with non-adherence. A preclinical assessment of semi-solid prodrug nanoparticle (SSPN) LAI formulations of emtricitabine (FTC) is reported here. Pharmacokinetics over 28 days were assessed in Wistar rats, New Zealand white rabbits, and Balb/C mice following intramuscular injection. Two lead formulations were assessed for the prevention of an HIV infection in NSG-cmah^−/−^ humanised mice to ensure antiviral activities were as anticipated according to the pharmacokinetics. Cmax was reached by 12, 48, and 24 h in rats, rabbits, and mice, respectively. Plasma concentrations were below the limit of detection (2 ng/mL) by 21 days in rats and rabbits, and 28 days in mice. Mice treated with SSPN formulations demonstrated undetectable viral loads (700 copies/mL detection limit), and HIV RNA remained undetectable 28 days post-infection in plasma, spleen, lung, and liver. The in vivo data presented here demonstrate that the combined prodrug/SSPN approach can provide a dramatically extended pharmacokinetic half-life across multiple preclinical species. Species differences in renal clearance of FTC mean that longer exposures are likely to be achievable in humans than in preclinical models.

## 1. Introduction

HIV (human immunodeficiency virus) infection is now commonly considered as a chronic disease rather than a terminal condition. In the absence of a cure, treatment requires a lifelong commitment to antiretroviral medication [1]. While there have been significant steps to reduce pill burden, and current treatment regimens can now be a single daily tablet, patient adherence to medication is an ongoing challenge over a life-time commitment to therapy [2].

Sub-optimal patient adherence has the potential to reduce efficacy and lead to treatment failure [3], and sub-therapeutic plasma exposure can allow continued viral replication and facilitate the development of mutations conferring drug resistance [4,5]. In some cases, a single mutation can confer class-wide resistance and compromises multiple agents in one event [6]. In addition to therapy, patient non-adherence has the potential to negatively impact pre-exposure prophylaxis (PrEP). Recently, mathematical simulations were used to explore the impact of not only reduced patient adherence but also patterns of adherence (simulations to predict efficacy following daily doses that were skipped periodically, randomly, or in large blocks). This study, along with other studies in HIV and schizophrenia, strongly indicates that pill-taking patterns exacerbate the reduction in efficacy [7,8,9,10].

Amongst the treatment strategies under development, long-acting injectable (LAI) formulations have received a great deal of interest. By increasing the dosing interval, the reliance on patient adherence is reduced. In addition to a clear need for alternative treatments, it has been demonstrated that patients would prefer the option of LAI. In a survey of 400 HIV-infected patients, 73% indicated they would definitely or probably try injectable nanoformulated antiretroviral therapy. Patient interest was demonstrated to increase with increasing the dosing interval; with 61%, 72%, and 84% interest in weekly, bi-weekly, and monthly dosing, respectively [11]. Patient preferences evaluations in clinical trials of existing LAI agents have also confirmed the high level of acceptability [12].

LAI formulations have been developed to address adherence issues in a number of other therapeutic areas [13,14]. Patient adherence has been a key driver to the development of LAI formulations, with ongoing research aiming to further increase the duration of therapy [14,15]. Given the proven utility in HIV and other treatment areas, interest in the development and deployment of LAI for HIV therapy and prevention is set to increase.

The recent approval of rilpivirine (RPV) and cabotegravir (CAB) for HIV treatment and/or prevention has further accelerated interest in LAI approaches for HIV [16]. Both RPV and CAB formulations provide plasma concentrations > 20 weeks in patients [17,18], although clinically they are used for shorter durations. Preclinical data describing tenofovir, maraviroc, and dolutegravir long-acting interventions have also emerged [19,20,21]. Nucleoside/nucleotide reverse transcriptase inhibitors (NRTIs) have conventionally been a core component of successful therapy and prevention [22].

Given the clear utility of NRTIs, an LAI formulation has the potential for application for the treatment and prevention of HIV. Accordingly, previous work described a unique prodrug/nanoparticle formation strategy to facilitate development of injectable aqueous semi-solid prodrug nanoparticle (SSPN) formulations of emtricitabine (FTC) [23]. The data presented herein describe the in vivo PK analysis in multiple preclinical species and antiviral activity of these SSPN LAI formulations of FTC in humanised mice.

## 2. Materials and Methods

### 2.1. Materials

Wistar rats, BALB/c mice, and New Zealand white rabbits were purchased from Charles River (Oxford, UK). NSG-cmah^−/−^ mice were bred at the University of Nebraska Medical Centre. Chloroform was purchased from Fisher Scientific (Loughborough, UK). FTC (>98% purity) was purchased from WISChem (Shanghai, China). All other consumables were purchased from Merck Life Science UK Ltd. (Dorset, UK).

### 2.2. Prodrug Synthesis

**Figure 1:**
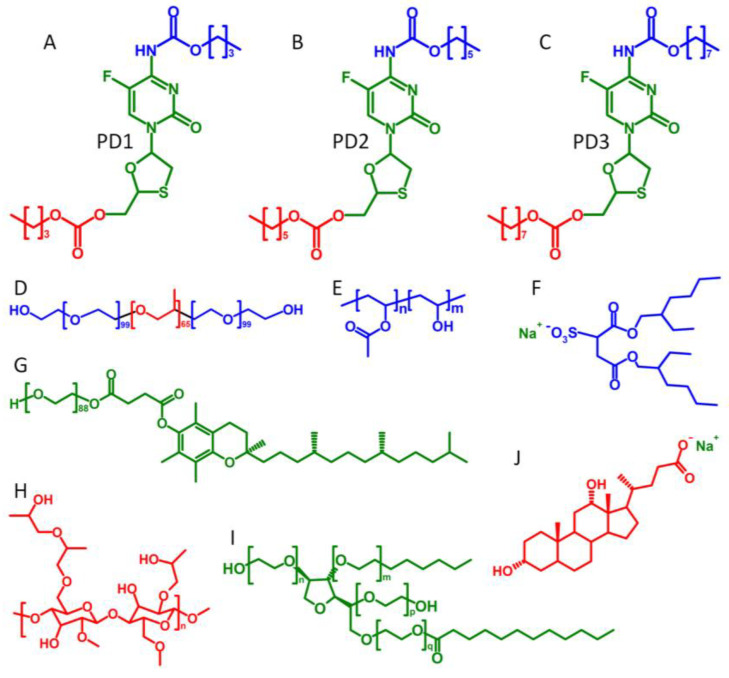
Structures of the varying chain length carbamate/carbonate FTC prodrugs, polymers, and surfactants used in SSPN manufacture: (**A**) prodrug 1, C_4_ derivatisation; (**B**) prodrug 2, C_6_ derivatisation; (**C**) prodrug 3, C_8_ derivatisation; (**D**) pluronic F127; (**E**) poly(vinyl alcohol) (PVA); (**F**) sodium dioctylsulfosuccinate (AOT); (**G**) d-alpha-tocopheryl polyethylene glycol succinate (TPGS); (**H**) hydroxypropyl methylcellulose (HPMC); (**I**) polysorbate-20 (Tween-20); and (**J**) sodium dexoxycholate (NDC).

Structures for the three FTC prodrugs investigated in this study are shown in Figure 1. FTC prodrugs were prepared using standard coupling conditions as previously described [23]. Briefly, in a flame-dried 25 mL round-bottom flask, cooled under argon, FTC (1.0 eq., 0.5 M) was suspended in DCM. Pyridine (3.0 eq., 1.5 M) was then added to the flask, and the resulting mixture was cooled to 0 °C in an ice-water bath. The reaction was initiated by the dropwise addition of the alkyl chloroformate (2.1 eq., 1.05 M). The reaction mixture was allowed to warm to room temperature with stirring. The reaction was deemed complete after 3 h, as monitored by TLC. Volatiles were removed from the reaction mixture under reduced pressure. The resulting residue was purified via silica flash chromatography (30% EtOAc in Hexane for 5 min, then 30–100% EtOAc over 6 min, then 100% EtOAc for 3 min).

### 2.3. SSPN Formulation

Polymers were weighed into glass vials and dissolved to a final concentration of 13.3 mg/mL (for 50 wt% loaded SSPNs) or 10 mg/mL (for 70 wt% loaded SSPNs), whilst surfactants were dissolved to 10 mg/mL (for 50 wt% loaded SSPNs) or 5 mg/mL (for 70 wt% loaded SSPNs) both in distilled water. Solutions were rolled overnight to ensure thorough dissolution. Each prodrug was stored at −20 °C and removed only immediately prior to SSPN synthesis, at a final concentration of 50 mg/mL or 70 mg/mL (for 50 wt% and 70 wt% loading, respectively) in chloroform. For SSPNs loaded with 50 wt% prodrug, 100 µL of surfactant, 300 µL of polymer, and 100 µL of prodrug was added to a 4 mL glass sample vial, whilst for SSPNs loaded with 70 wt% prodrug, 200 µL of surfactant, 200 µL of polymer, and 100 µL of prodrug was added to a 4 mL glass vial. This emulsion consisting of chloroform-in-water was sonicated for 15s using a Covaris S220X, and then immediately transferred to liquid nitrogen to ensure rapid freezing. The frozen samples were placed into a VirTis BenchTop K freeze dryer (SP Scientific, Ipswich, UK) set to −100 °C and vacuum of <40 μbar for 48 h, after which they were kept in an ambient temperature desiccator. The compositions of the formulations used in the current study are shown in Table 1.

### 2.4. Pharmacokinetics of SSPN Formulations in Wistar Rats

Wistar rats, New Zealand white rabbits, and BALB/c mice (Charles River UK) were used for PK analysis of FTC. PK experiments were conducted in accordance with the Animals (Scientific Procedures) Act 1986 (ASPA), implemented by the United Kingdom Home Office. NSG-cmah^−/−^ were obtained from the established breeding colony and housed under pathogen-free conditions in accordance with ethical guidelines for care of laboratory animals at the National Institutes of Health and the University of Nebraska Medical Centre.

An initial screen of 12 candidate formulations (Table 1) was conducted in Wistar rats. Adult male Wistar rats (~300 g) were randomised into 12 groups, (N = 1 per group). Groups were dosed with SSPN formulations at 10 mg/kg (FTC equivalent) via a single intramuscular (IM) injection in the musculus biceps femoris (0.1 mL volume). Blood samples were collected (500 µL) post-dosing from the tail vein every 24 h over 7 days and stored at −80 °C until further analysis. At the point of termination, the rats were sacrificed using a rising gradient of CO_2_ followed by cervical dislocation. Plasma concentrations of FTC were quantified using LC-MS/MS.

Three lead formulations (Table 1; SSPN-9, -10 and -12) were selected on the basis of PK performance in screening. These formulations were progressed to a longer-term study in adult male Wistar rats (~300 g). Animals were randomised into 3 groups (*n* = 3 per group), and dosed with SSPN formulations at 40 mg/Kg (FTC equivalent) via 2 IM injections in both the left and right musculus biceps femoris (0.1 mL volume per site). Blood samples (100 µL) were collected at 1.5, 3, 6, 24, 48, 96, 168, 240, 336, 504, and 672 h from the tail vein and stored at −80 °C until further analysis. At the point of termination, the rats were sacrificed using a rising gradient of CO_2_ followed by cervical dislocation. Plasma concentrations of FTC were quantified using LC-MS/MS as described below.

### 2.5. Pharmacokinetics of Lead SSPN Formulations in New Zealand White Rabbits

The three lead formulations (Table 1 SSPN-9, -10 and -12) were further characterised in adult male New Zealand white rabbits (~3 kg), randomised into 3 groups, (*n* = 3 per group). Groups were dosed with SSPN formulations at 40 mg/Kg (FTC equivalent) via 2 IM injections in both the left and right musculus biceps femoris (1 mL volume per site). Blood samples (500 µL) were collected at 1.5, 3, 6, 24, 48, 96, 168, 240, 336, 408, 504, 576, and 672 h from the marginal ear vein and stored at −80 °C until further analysis. At the point of termination, the rabbits were sacrificed via intra-venous injection of an anaesthetic agent. Plasma concentrations of FTC were quantified using LC-MS/MS as described below.

### 2.6. Pharmacokinetics of Lead SSPN Formulations in BALB/c Mice

SSPN-9 and -10 were selected for pharmacokinetic and efficacy studies in mice because they enabled the evaluation of near identical formulations for two different prodrugs. Balb/C mice (aged 11–12 weeks, ~25 g), were randomised into 3 groups, (*n* = 3 per group). Groups were dosed with SSPN formulations at 140 mg/Kg (FTC equivalent) via 2 IM injections in both the left and right musculus biceps femoris (50 µL volume per site). A serial sacrifice experimental design was utilised. Mice were sacrificed at each time point via rising CO_2_ gradient. Blood was collected post-mortem via cardiac puncture and transferred to heparinised sample tubes. Samples were collected at 1.5, 3, 6, 24, 48, 96, 168, 240, 336, 504, and 672 h and stored at −80 °C until further analysis. Plasma concentrations of FTC were quantified using LC-MS/MS as described below.

### 2.7. Quantification of FTC in Plasma

FTC was detected using a fully validated method adapted from [24]. Quantification was achieved via LC-MS/MS (TSQ Endura, Thermo Scientific, Waltham, MA, USA) operating in positive mode. The following ions were monitored for quantification in selected reaction monitoring scan: FTC (*m*/*z* 248 > 113 and 130). A stock solution of 1 mg/mL FTC was prepared in H_2_O and stored at 4 °C until use. A standard curve was prepared in plasma by serial dilution from 500 ng/mL to 1.9 ng/mL and an additional blank solution. Chromatographic separation was achieved using a multi-step gradient with a Synergi polar C18 column (4 mm:150 mm:2.0 mm; Phenomenex, Cheshire, UK) using mobile phases A (100% H_2_O, 0.1% formic acid) and B (100% ACN, 0.1% formic acid). Chromatography was conducted over 6 min at a flow rate of 400 µL/min. At the start of each run, mobile phase A was 95% until 0.2 min when mobile phase B was increased to 30% at 0.5 min. Mobile phase B was then maintained at 30% until 3 min. Mobile phase B was then reduced to 5% at 3.2 min which was held until 6 min. Inter- and intra-assay variance was assessed by 3 levels of independent standards. The coefficient of variation of accuracy and precision were <15% in all assays (Supplementary Materials Table S1).

### 2.8. Pharmacokinetic Modelling in Rats, Rabbits, and Mice

Plasma concentration–time profiles from all three species were each fitted with a one-compartment disposition PK model with first order absorption input from depot release using the R data analysis environment (v 4.1.1) [25], treating the composite PK profile from all rats, rabbits, or mice for a given treatment as a naïve pool. Fittings made use of the Pracma library [26] and the lsqnonlin function for nonlinear regression. Apparent clearance (CL/F), apparent volume of distribution (V/F), and absorption rate constant (KA) parameters were estimated in the absence of IV data to quantify absolute bioavailability in each species in this study.

### 2.9. Efficacy of SSPN Formulations in NSG-Cmah^−/−^ Mice

A human immune system was reconstituted in NSG-cmah^−/−^ mice, as previously described [27,28]. In brief, mouse pups (aged 0–4 days) first received full body irradiation (1Gy). CD34+ human stem cells (5 × 10^4^ cells) were then delivered via intra-hepatic injection 6 h post-irradiation. Reconstitution of the human immune cells was monitored via flow cytometry (anti-human CD45-FITC BD Pharmigen, #555482, CD3-Alexa Fluor 700 BD Biosciences 557943, CD4-APC BD Pharmingen #555349, CD8-BV421 BD Horizons #562428). Humanised NSG-cmah^−/−^ mice were then used to investigate the efficacy of SSPN formulations when applied to PrEP. Humanised mice were randomised into 2 groups: a 7-day challenge and a 14-day challenge. Each group was subdivided into 3 treatment conditions (8 mice per treatment): untreated control, SSPN-9, and SSPN-10 (Table 1). Mice were treated at day 0 with SSPN-9 or SSPN-10 (140 mg/Kg FTC equivalent) via 2 IM injections in the left and right musculus biceps femoris (50 µL volume per site). HIV-1_ADA_ challenge was administered 7- or 14-days post-drug administration via intraperitoneal injection (10^4^ 50% tissue culture infectious dose; TCID_50_). Blood samples were taken at 14 days post-infection to determine viral load via PCR (COBAS^®^ AmpliPrep TaqMan^®^ HIV-1 Test, v2.0, Roche Molecular Systems Inc., Pleasanton, CA, USA). All animals were terminated at 28 days post-infection. Plasma samples were collected for determination of viral load via PCR (COBAS^®^ AmpliPrep TaqMan^®^ HIV-1 Test, v2.0, Roche Molecular Systems Inc., Pleasanton, CA, USA) and characterisation of human immune cells by flow cytometry. The acquisition was conducted on BD LSRII flow cytometer cells and analysed on gated leukocytes using FLOJO analysis software (v 10.2 Tree Star, Ashland, OR, USA).

Following termination, samples from spleen, liver, and lung tissues were collected and stored at −80 °C until analysed. Tissue processing was carried out as previously described [19]. In brief, tissue samples were homogenised using a bead mill homogeniser (Qiagen TissueLyzer II Valencia, CA, USA) and Qiagen AllPrep DNA/RNA mini kit (Hilden, Germany). cDNA was produced from RNA using ThermoScientific Verso cDNA synthesis kit (Vilnius, Lithuania) as per manufacturer’s instructions. RT-PCR HIV-1 Gag p24 was conducted using primers and probe assays purchased from Life Technologies (forward primer ACATCAAGCAGCCATGCAAAT, reverse primer ATCTGGCCTGGTGCAATAGG, probe sequence 6FAMCATCAATGAGGAAGCTGCAGAATGGGATAGATAMRA). Thermal cycling was conducted using a QuantStudio™ 3 system (Applied Biosystems^®^, Waltham, MA, USA) programmed for standard cycling conditions (95 °C for 10 min, followed by 40 cycles of 95 °C for 15 s, and 50 °C for 1 min).

Following termination, samples from spleen, liver, and lung tissue were collected and fixed in paraformaldehyde (4%). The 5 µm sections were collected and stained for HIV-1 p24 (1:10, DAKO, Carpinteria, CA, USA) or HLA-DQ/DP/DR (clone CR3/43, 1:100, DAKO, Carpinteria, CA, USA) and incubated overnight at 4 °C as previously described [29]. Tissue samples were washed 3× in TBS-Tween and then incubated with secondary reagent the polymer-based horse radish peroxidase (HRP) and 3,3′-Diaminobenzidine (DAB) DAKO EnVision system for 15 min. Tissue samples were counterstained in filtered hematoxylin for 1 min. Cells were visualised at 20× and 40× on a Nuance EX camera fixed to a Nikon Eclipse E800 microscope using Nuance software (Cambridge Research & Instrumentation, Woburn, MA, USA).

Differences in viral load in plasma and tissues at timepoints 14 and 28 days following challenge 7 or 14 days after dosing of SDN9, SDN10, or control/no treatment were tested for significance using a one-sided, pairwise Mann–Whitney–Wilcoxon test, with a Bonferroni correction factor of 3 (for 3 pairwise comparison tests per challenge/tissue/timepoint group).

## 3. Results

### 3.1. Characterisation of Formulations Used in the Studies

**Figure 2:**
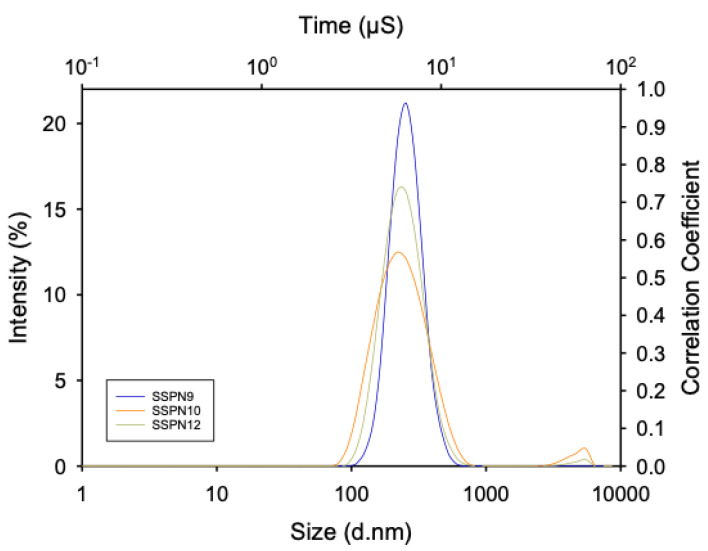
Representative z-average hydrodynamic diameter particle size distribution of SSPN−9 (blue), SSPN-10 (orange), and SSPN-12 (yellow). Data are shown as the average of 3 measurements from dynamic light scattering analysis.

All of the SSPN formulations displayed narrow, monomodal particle size distributions (Figure 2) with z-average (Dz) diameters ranging from 196 to 782 nm and polydispersity index (PDI) values varying from 0.295 to 0.450 across the three different prodrugs (Table 1). These criteria were selected as uniformity was expected to confer reproducible drug release.

### 3.2. Initial Pharmacokinetic Screening of SSPN Formulations in Wistar Rats

An initial screen of 12 candidate formulations (Table 1) comprising 3 FTC prodrugs (PDs) and dual combinations of three polymers and four surfactants were tested for in vivo PK in Wistar rats. Each candidate was administered via a single IM injection (10 mg/kg based on FTC content, *n* = 1). Plasma samples were collected daily for 7 days (Table 2). SSPN 1–8 demonstrated poor plasma exposure, Cmax ranged between 19.8 ng/mL and <2 ng/mL. Plasma concentrations in all eight formulations fell below the limit of detection (2 ng/mL) within 4 days. SSPN-11 demonstrated high burst release (Cmax 88.5 ng/mL) before FTC concentrations rapidly decreased to 2.45 ng/mL by day 7. The remaining 3 formulations (SSPN-9, -10, and -12) all demonstrated favourable PK for LAI, Cmax (SSPN-9 42.8 ng/mL, SSPN-10 33.9 ng/mL, and SSPN-12 47.1 ng/mL), and Cmin (SSPN-9 22.2 ng/mL, SSPN-10 24.1 ng/mL, and SSPN-12 16.9 ng/mL) over 7 days. SSPN formulations SSPN-9, SSPN-10, and SSPN-12 were therefore progressed to a longer-term study.

### 3.3. Longer-Term Pharmacokinetic Evaluation of Lead SSPN Formulations in Wistar Rats

**Figure 3:**
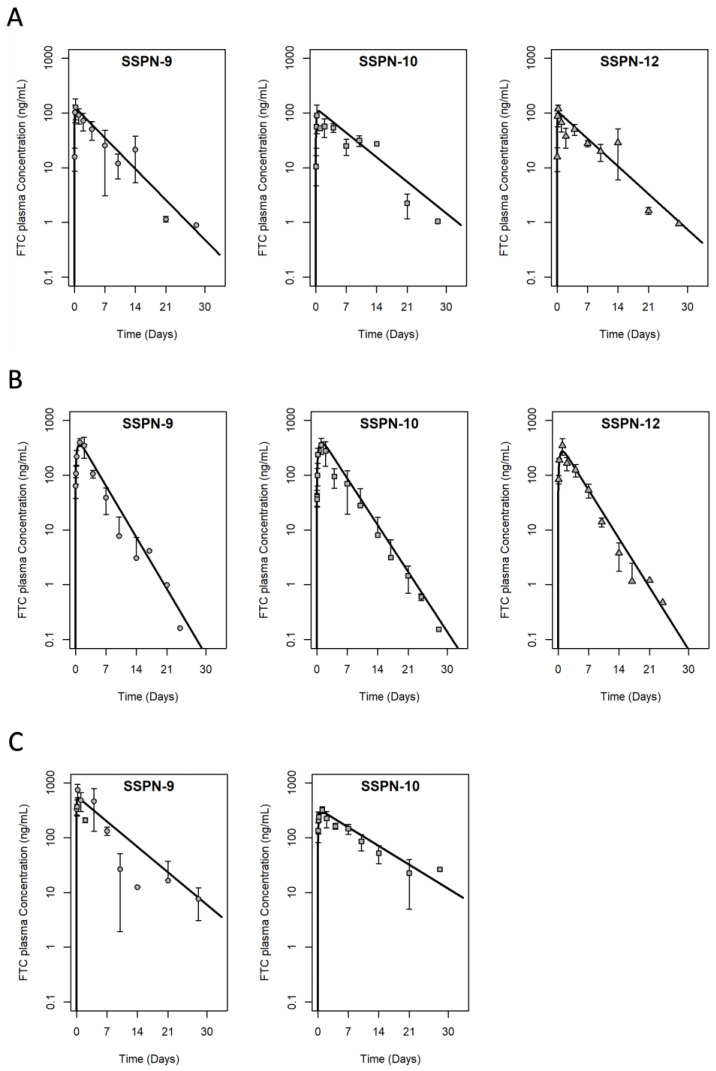
PK profiles of the three lead SSPN candidates over 28 days in (**A**) Wistar rats (**B**) New Zealand white rabbits, and (**C**) Balb/C mice. Animals were administered 2 IM injections (1 injection in each leg, given simultaneously) of 40 mg/kg for Wistar rats, 40 mg/kg for New Zealand white rabbits, and 140 mg/kg for Balb/C mice based on FTC content. Error bars represent standard deviation. Solid line is the PK model fitting to the naïve pooled data (1-compartment disposition with 1st order depot release input).

Three lead formulations (SSPN-9, -10, and -12 derived from C6 and C8 FTC-PDs) were progressed to a longer-term study. Each candidate was administered via 2 IM injections (20 mg/kg each = 40 mg/kg total dose based on FTC content, *n* = 3) and plasma samples were collected over 28 days (Figure 3A). Cmax was reached by 6–12 h (SSPN-9 127 ± 52.9 ng/mL, SSPN-10 95 ± 40.6 ng/mL, and SSPN-12 119 ± 21.0 ng/mL) and plasma concentrations were detectable for all three formulations until 14 days (SSPN-9 21 ± 16.2 ng/mL, SSPN-10 27 ± 0.05 ng/mL, and SSPN-12 29 ± 22.7 ng/mL). Plasma concentrations were below the validated limit of detection (2 ng/mL) by 21 days for all three formulations (Figure 3A, Table 3). The rat data were adequately described by a one-compartment disposition PK model with first order depot release input, with acceptable precision of parameter estimates. As expected for this formulation, “flip-flop” PK was demonstrated [30] where the observed, long terminal phase half-life of the profiles (T1/2, SSPN-9 89 h, SSPN-10 113 h, and SSPN-12 100 h) reflects the kinetics of slow release from the depot rather than the elimination rate constant which is intrinsic to the disposition of FTC as shown following IV dose [31]. This is also reflected in the parameter estimates where the absorption rate constant KA is smaller (and therefore slower) than elimination rate constant as calculated from CL divided by V.

### 3.4. Long-Acting PK Study in New Zealand White Rabbits

Multiple species are employed in preclinical screening due to species differences dictating differences in drug exposure. The confirmation of LAI plasma exposure was, therefore, also evaluated in rabbits for lead formulations. Each candidate was administered via 2 IM injections (20 mg/kg each = 40 mg/kg total dose based on FTC content, *n* = 3) and plasma samples were collected over 28 days (Figure 3B). Cmax was reached at 24–48 h (SSPN-9 416 ± 93.6 ng/mL, SSPN-10 356 ± 116.9 ng/mL, and SSPN-12 348 ± 117.3 ng/mL) and plasma concentrations were detectable for all three formulations until 14 days (SSPN-9 3.1 ± 4.3 ng/mL, SSPN-10 8 ± 9.0 ng/mL, and SSPN-12 3.8 ± 2.0 ng/mL). Plasma concentrations were below the validated limit of detection (2 ng/mL) by 21 days for all three formulations (Figure 3B, Table 3). As with the rat data, the rabbit plasma PK data were adequately described by a one-compartment disposition model with first order depot release input, with acceptable precision of parameter estimates. Flip-flop PK was again demonstrated with a slow KA estimate and slow terminal half-life; however, for all three formulations, this terminal phase was ~2× faster in rabbits than in rats, implying a faster release from the depot in rabbits than rats (T1/2, SSPN-9 54 h, SSPN-10 59 h, and SSPN-12 57 h).

### 3.5. Long-Acting PK Study in BALB/c Mice

**Figure 4:**
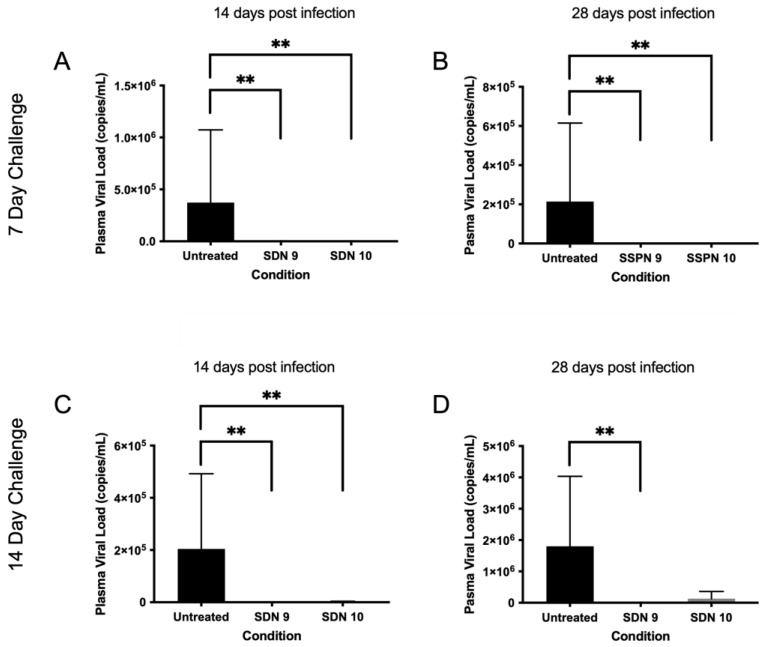
Plasma viral load following HIV challenge 7 (**A**,**B**) and 14 days (**C**,**D**) post-injection. Subfigures show viral load (copies/mL) at 14- and 28-days post-infection IM injection (700 copies/mL detection limit). ** denotes *p* value > 0.01 determined using significance using a one-sided, pairwise Mann–Whitney–Wilcoxon test, with Bonferroni correction factor.

Two candidates were selected for efficacy evaluation in humanised mice and supportive pharmacokinetic evaluation was conducted ahead of efficacy studies in Balb/c mice. For this, SSPN-9 and -10 were selected because they enabled evaluation of near identical formulations for two different prodrugs. Each candidate was administered via 2 IM injections (70 mg/kg each = 140 mg/kg total dose based on FTC content, *n* = 3) and plasma samples were collected over 28 days (Figure 4C). Cmax was reached within 24 h (SSPN-9 747 ± 206.5 ng/mL and SSPN-10 330 ± 41.5 ng/mL) and plasma concentrations were detectable for all three formulations until 28 days (SSPN-9 7.7 ± 4.6 ng/mL and SSPN-10 26.6 ± 0.63 ng/mL) (Figure 4C, Table 3). As with the rat and rabbit, the mouse plasma PK data were adequately described by a one-compartment disposition model with first order depot release input, with acceptable precision of parameter estimates. Again, flip-flop PK is demonstrated with a slow KA estimate, which is further reflected in the slow terminal half-life, which is more similar in magnitude to the rat than the rabbit for the two lead formulations examined (T1/2, SSPN-9 109 h, SSPN-10 148 h).

### 3.6. In Vivo Protection against HIV-1 Infection by Lead SSPN Formulations

**Figure 5:**
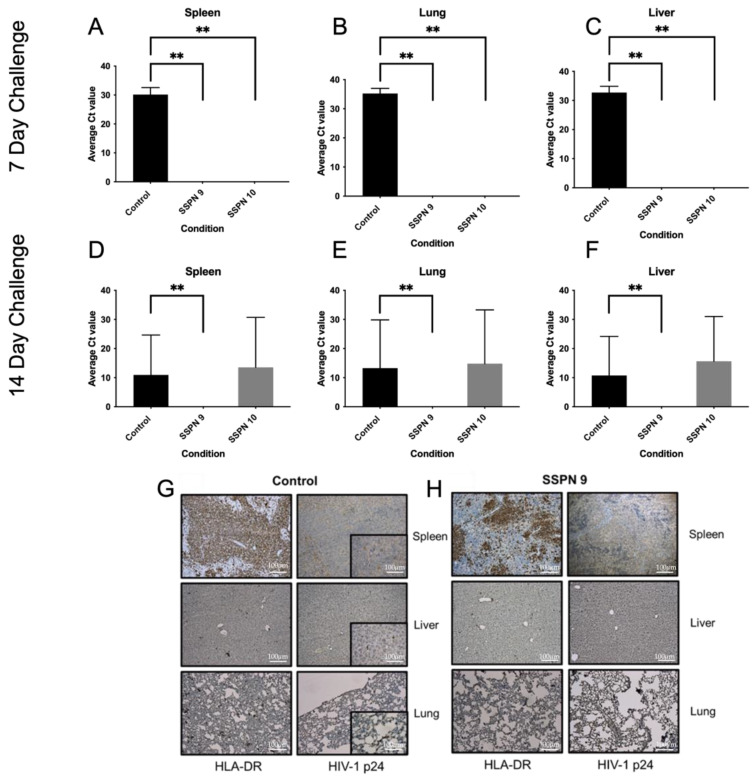
This table shows detection of HIV-1 Gag-p24 RNA via PCR in humanised mice following HIV infection 7 (**A**–**C**) and 14 (**D**–**F**) days post-IM injection. Tissue samples from spleen, lung, and liver were taken 28 days post-HIV infection. Also shown are representative histopathology samples taken from spleen, liver, and lung tissues 7 days post-IM injection from the (**G**) control group and (**H**) SSPN-9-treated group. Tissue samples were collected 28 days post-HIV infection. Samples were stained for HLA-DR and Gag-p24. Gag-p24-positive staining in the control group (brown). No Gag-p24 was detected in the SSPN-9-treated group. ** denotes *p* value > 0.01 determined using significance using a one-sided, pairwise Mann–Whitney–Wilcoxon test, with Bonferroni correction factor.

In order to assess the efficacy of the SSPN formulations in preventing an HIV infection, a humanised mouse model of HIV infection was employed. Humanised mice were divided into 2 groups: a 7-day challenge and a 14-day challenge. Humanised mice were treated at day 0 with SSPN-9 or SSPN-10 (140 mg/Kg FTC equivalent) via 2 IM injections. Virologic challenge was administered 7- or 14-days post-drug administration (10^4^ TCID_50_). Plasma viral load was undetectable in mice treated with SSPN-9 and SSPN-10 VL (700 copies/mL detection limit) 14 days and 28 days post-infection (Figure 4A,B). HIV RNA was also undetectable 28 days post-infection in spleen, lung, and liver tissue in all animals for the 7-day challenge (Figure 5A–C). Further tissue samples were stained for HLA- DR (human leukocyte surface antigen) and Gag-p24 (HIV capsid protein). The histopathology of these samples confirmed the presence of human leucocytes while confirming undetectable viral protein in the SSPN treatment groups (Figure 5G,H).

Following the 14-day challenge, mice treated with SSPN-9 demonstrated undetectable HIV in plasma 14 days and 28 days post-infection (Figure 4C,D). Viral RNA was also undetectable 28 days post-infection in spleen, lung, and liver tissue (Figure 5D–F). Mice treated with SSPN-10 demonstrated that 2 mice had detectable plasma VL (4.77 × 10^3^ copies/mL) and 3 mice showed presence of HIV RNA in plasma and proteins in spleen, lung, and liver on day 28 (Figure 5D–F). HIV was detectable in all untreated animals. *p*-values summarising the significance of the differences in VL between SDN9 and SDN10 treatment vs. untreated following 7- and 14-day viral challenges are broadly consistent with visual presentation of the data in Figure 4 and Figure 5 (Supplementary Materials Table S2). For a day 7 challenge post-dose, both SDN9 and SDN10 treatment give significantly lower plasma VL than untreated (and are not significantly different from each other) both 14 and 28 days post-challenge infection. SDN9 treatment also has significantly lower VL in plasma and tissues vs. untreated at both 14 and 28 days post-infection after a day 14 challenge post-dose. Due to greater inter-animal variability in VL following a 14-day challenge after SDN10 dosing, *p*-values indicate greater ambiguity in distinguishing SDN10 treatment from both untreated and the SDN9-treated groups.

## 4. Discussion

Existing antiretroviral LAI utilise milling to produce particle dispersions where controlled release is achieved by slow dissolution as a result of low drug solubility. As with many NRTI’s, FTC is highly water-soluble, making it incompatible with technologies such as milling. In order to circumvent this limitation, three varying chain-length carbamate/carbonate prodrugs of FTC were produced to mask key hydrophilic groups (Figure 1) to decrease aqueous solubility. These highly lipophilic prodrugs are semi-solid at ambient temperatures and are thus also not compatible with bottom-down processing technologies such as nanomilling. However, compatibility with emulsion-templated freeze drying (ETFD) has been explicitly demonstrated to form particle dispersions from this PD class via this approach [23]. The in vivo data presented here demonstrate that the SSPN approach can provide a half-life extension for water-soluble FTC in multiple pre-clinical species. This approach represents an interesting avenue of research to allow the exploitation of antiretrovirals that would otherwise be incompatible for processing or formulation with LAI.

In order for FTC to exert efficacy, it must be taken up and intracellularly undergo phosphorylation to the active FTC-TP. In the current study, intracellular concentrations of FTC-TP were not examined. Guidelines for the treatment of laboratory animals limit the sample volumes that may be taken over the experimental period, precluding the option to assay for both FTC and FTC-TP in mice and rats [32,33]. However, it can be concluded that the SSPN formulations successfully deliver FTC without abrogation of phosphorylation from the demonstration of the effectiveness in the mouse model of HIV infection. The plasma half-life in humans of FTC following oral administration is 7.4 h, whereas the intracellular half-life of FTC-TP is 39 h [34,35]. The active FTC-TP is present in vivo over 5 times as long as FTC, indicating plasma concentrations alone may underestimate the efficacy of the LAI formulations presented here. The lead formulation (SSPN-9) presented here demonstrated an 18.5-fold (4.79 h vs. 89 h) and 19.5-fold (5.6 h vs. 109 h) plasma half-life extension with SSPN compared to oral administration in mice and rats, respectively [31,36]. No published pharmacokinetics data for orally administered FTC are available in rabbits, but consistency in the half-life extension between mouse and rat is reassuring. The oral plasma half-life of FTC in humans is approximately 10 h, and the presented data therefore support a half-life longer than 185 h for SSPN-9 in humans if the relationship holds.

The data presented here show some clear patterns in the SSPN composition and PK exposure. Amongst the excipients tested, the formulations that performed optimally contained HPMC (with either AOT or NDC). The importance of including HPMC is not entirely unexpected as HPMC has been used frequently to modulate drug release from oral formulations [37]. In addition to the selection of polymer and surfactant, the inclusion of different prodrugs appears to affect the release profile. Formulations SSPN-11 and SSPN-12 both consist of HPMC and AOT but differ in prodrug composition. SSPN-11 contained C6 carbamate/carbonate while SSPN-12 contained the C8 carbamate/carbonate prodrug. Despite the similar formulation, the resulting PK behaviour differed. SSPN-12 demonstrated a modulated steady release, providing plasma concentrations up to 14 days; whereas, SSPN-11 showed a high burst release followed by rapid decrease in plasma exposures. Interestingly, this relationship was not observed when PK for SSPN-9 and SSPN-10 were compared. Both formulations comprised HPMC with NDC and either C6 or C8 carbamate/carbonate and show very similar PK profiles. These data demonstrate the careful consideration required when selecting polymer, surfactant, and active pharmaceutical agent, as the performance may change in an unpredictable manner.

While the current data do not enable robust conclusions to be drawn about how prodrug structures and formulation compositions might impact PK performance, both formulations studied in humanised mice were able to protect from the acquisition of an HIV infection. Importantly, there were differences in PK exposure between SSPN-9 and SSPN-10, with a higher C_max_ and AUC observed for SSPN-9 in Balb/c mice. In agreement with these PK observations, the duration of protection for SSPN-9 was longer than that for SSPN-10, validating the pharmacological strategy for selection of lead candidates. SSPN-9 and SSPN-10 differed only regarding the prodrug contained therein, and both formulations were composed of HPMC and NDC, with similar Z-average diameter and PDI. It is, therefore, reasonable to conclude that the chain length of the carbamate/carbonate prodrug moiety is an important contributor to overall performance of the SSPN formulation.

The development of LAI is hindered by the paucity of knowledge of the mechanisms involved in the release from the depot. Many of the physiological, anatomical, and formulation-specific characteristics involved are poorly characterised, making targeting successful LAI problematic. Recent data indicate several mechanisms within the depot site that may contribute to LAI PK performance. IM administration of paliperidone palmitate has been shown to illicit a granulomatous response with macrophage penetration. Furthermore, co-administration of sunitinib completely suppressed the granulomatous reaction and effectively inhibited the neovascularization of the paliperidone palmitate LAI depot. This resulted in a slower systemic exposure with delayed and lower C_max_ [38]. As a better understanding of depot site mechanisms emerges, it may be possible to tune prodrug and formulation composition towards critical mechanisms that optimize LAI behaviour.

## 5. Conclusions

The data presented here show the successful in vivo application of SSPN-based LAI to provide half-life extension for lipophilic bio-reversible PDs of water-soluble drugs. Species differences in clearance would indicate that the formulations in humans may provide even greater exposure. In general, smaller species have a higher rate of clearance [39]. FTC specifically is renally cleared at a rate of 22.3 mL/kg/min in rats and 3.5 mL/kg/min in humans [31,40]. The difference in clearance would indicate the potential for plasma concentrations of FTC to remain in circulation longer in humans than those observed in preclinical species.

This formulation strategy shows the potential to add a new class of antiretrovirals to LAI treatment or prevention options. In addition to potential applications towards co-formulation with other antiretrovirals, LAI FTC may have applications in PrEP. Future work will aim to assess the applicability of this approach to other water-soluble drugs for HIV and other indications.

## Figures and Tables

**Table 1:** This table shows the formulation number, excipients (polymer and surfactant), prodrug designation percentage drug loading, and physical characterisation parameters.

Formulation	Polymer	Surfactant	Prodrug	Prodrug Loading (wt%)	Z-Average nm (±SD)	PDI (±SD)
SSPN-1	F127	TPGS	PD3	70	267 (26)	0.314 (0.022)
SSPN-2	PVA	Tween 20	PD1	70	412 (15.5)	0.347 (0.045)
SSPN-3	F127	TPGS	PD1	50	702 (111)	0.305 (0.090)
SSPN-4	F127	TPGS	PD2	50	782 (199)	0.450 (0.152)
SSPN-5	F127	TPGS	PD3	50	239 (50)	0.323 (0.042)
SSPN-6	PVA	Tween 20	PD1	50	298 (21)	0.339 (0.038)
SSPN-7	PVA	Tween 20	PD2	50	261 (44)	0.295 (0.019)
SSPN-8	PVA	Tween 20	PD3	50	293 (9)	0.294 (0.028)
SSPN-9	HPMC	NDC	PD2	50	196 (6)	0.345 (0.027)
SSPN-10	HPMC	NDC	PD3	50	261 (17)	0.376 (0.038)
SSPN-11	HPMC	AOT	PD2	50	217 (10)	0.306 (0.022)
SSPN-12	HPMC	AOT	PD3	50	237 (5)	0.296 (0.021)

**Table 2:** This table shows the 12 SSPN candidates. The table shows PK parameters (Cmax, Tmax, and AUC) over 7 days following a single IM injection (10 mg/kg based on FTC content).

Formulation	C_max_ (ng/mL)	C_min_ (ng/mL)	T_max_ (h)	AUC (ng/h/mL)
SSPN-1	2.3	<2.0	72	170.5
SSPN-2	<2.0	<2.0	24	89.0
SSPN-3	<2.0	<2.0	24	86.0
SSPN-4	19.8	<2.0	24	802.3
SSPN-5	6.0	<2.0	48	305.9
SSPN-6	3.5	<2.0	24	236.1
SSPN-7	12.1	<2.0	24	443.2
SSPN-8	3.3	<2.0	72	169.1
SSPN-9	42.8	22.2	24	3449.7
SSPN-10	33.9	24.1	24	3731.0
SSPN-11	88.5	2.5	24	3960.9
SSPN-12	47.1	16.9	24	3695.1

**Table 3:** PK parameters for the three lead SSPN candidates in Wistar rats, New Zealand white rabbits, and Balb/C mice (Cmax [maximum plasma concentration], C 14 [plasma concentration at day 14], Tmax [time maximum plasma concentration was reached], AUC [area under the curve], CL/F [clearance], V/F [volume of distribution], KA [absorption rate constant], and T_1/2_ [terminal half-life] from the PK model fitting) over 28 days following 2 IM injections (1 injection in each leg, given simultaneously, 40 mg/kg total dose based on FTC content). SD = standard deviation; % RSE = “% relative standard error of estimate”.

Formulation		C_max_	C_14_	T_max_	AUC	CL/F	V/F	KA	Terminal T_1/2_
	Species	(ng/mL) (SD)	(ng/mL) (SD)	(h) (SD)	(ng·h/mL) (SD)	(L/h/kg) (% RSE)	(L/kg) (% RSE)	(h^−1^) (% RSE)	(h)
SSPN-9	Wistar rat	127 (52.9)	21 (16.2)	6 (0.0)	15,136 (6306.1)	2.44 (11)	5.02 (38)	0.0078 (7)	89
	NZW rabbit	416 (93.6)	3.1 (4.3)	32 (13.9)	33,680 (8817.7)	1.05 (4)	10 (1)	0.0129 (6)	54
	Balb/C mice	747 (206.5)	7.7 (4.6)	6 (0.0)	60,387 (21,111.2)	1.56 (12)	2.38 (55)	0.0064 (9)	109
SSPN-10	Wistar rat	95 (40.6)	27 (0.1)	12 (10.4)	16,003 (3220.3)	2.06 (12)	8.72 (30)	0.0061 (8)	113
	NZW rabbit	356 (116.9)	8 (9.0)	24 (0.0)	34,444 (5587.3)	0.87 (10)	10.07 (12)	0.0117 (4)	59
	Balb/C mice	330 (41.5)	26.6 (0.63)	17 (12.1)	57,870 (11,698.8)	1.97 (10)	9.99 (27)	0.0047 (11)	148
SSPN-12	Wistar rat	119 (21.0)	29 (22.7)	6 (0.0)	15,096 (3208.3)	2.58 (9)	5.30 (35)	0.0069 (6)	100
	NZW rabbit	348 (117.3)	3.8 (2.0)	24 (0.0)	28,414 (4674.4)	1.33 (4)	12.85 (11)	0.0122 (4)	57
	Balb/C mice	-	-	-	-	-	-	-	-

## Data Availability

Data available on request due to restrictions privacy and intellectual property. The data presented in this study are available on request from the corresponding author. The data are not publicly available due to privacy restrictions.

## Supplementary Materials

The following supporting information can be downloaded at: https://www.mdpi.com/article/10.3390/pharmaceutics15071835/s1, Table S1 Accuracy and precision of the validated LCMS assay. Table S2 Statistical assessment for significance of Protection against HIV-1 Infection by Lead SSPN Formulation.

## References

[1] Bhatti A.B., Usman M., Kandi V. (2016). Current Scenario of HIV/AIDS, Treatment Options, and Major Challenges with Compliance to Antiretroviral Therapy. Cureus.

[2] Landovitz R.J., Grinsztejn B. (2016). Long-Acting Injectable Preexposure Prophylaxis for HIV Prevention in South Africa: Is There a Will and a Way?. J. Infect. Dis..

[3] Rosenbloom D.I., Hill A.L., Rabi S.A., Siliciano R.F., Nowak M.A. (2012). Antiretroviral dynamics determines HIV evolution and predicts therapy outcome. Nat. Med..

[4] Ssemwanga D., Lihana R.W., Ugoji C., Abimiku A., Nkengasong J., Dakum P., Ndembi N. (2014). Update on HIV-1 Acquired and Transmitted Drug Resistance in Africa. AIDS Rev..

[5] Abram M.E., Ferris A.L., Das K., Quinones O., Shao W., Tuske S., Alvord W.G., Arnold E., Hughes S.H. (2014). Mutations in HIV-1 reverse transcriptase affect the errors made in a single cycle of viral replication. J. Virol..

[6] Luber A.D. (2005). Genetic barriers to resistance and impact on clinical response. J. Int. AIDS Soc. Vol..

[7] Dimitrov D.T., Masse B.R., Donnell D. (2016). PrEP Adherence Patterns Strongly Affect Individual HIV Risk and Observed Efficacy in Randomized Clinical Trials. J. Acquir. Immune Defic. Syndr..

[8] Scarsi K.K., Swindells S. (2021). The Promise of Improved Adherence with Long-Acting Antiretroviral Therapy: What Are the Data?. J. Int. Assoc. Provid. AIDS Care.

[9] D’Amico R., Cenoz Gomis S., Moodley R., Van Solingen-Ristea R., Baugh B., Van Landuyt E., Van Eygen V., Min S., Cutrell A., Foster C. (2023). Compassionate use of long-acting cabotegravir plus rilpivirine for people living with HIV-1 in need of parenteral antiretroviral therapy. HIV Med..

[10] Romagnoli A., Santoleri F., Costantini A. (2021). Long-acting Injectable vs. Oral Antipsychotics: Adherence, Persistence and Switching over three Years of Real-life Analysis. Curr. Rev. Clin. Exp. Pharm..

[11] Williams J., Sayles H.R., Meza J.L., Sayre P., Sandkovsky U., Gendelman H.E., Flexner C., Swindells S. (2013). Long-acting parenteral nanoformulated antiretroviral therapy: Interest and attitudes of HIV-infected patients. Nanomedicine.

[12] Titus-Lay E.N., Ansara E.D., Isaacs A.N., Ott C.A. (2018). Evaluation of adherence and persistence with oral versus long-acting injectable antipsychotics in patients with early psychosis. Ment. Health Clin..

[13] Brissos S., Veguilla M.R., Taylor D., Balanza-Martinez V. (2014). The role of long-acting injectable antipsychotics in schizophrenia: A critical appraisal. Adv. Psychopharmacol..

[14] Tolley E.E., McKenna K., Mackenzie C., Ngabo F., Munyambanza E., Arcara J., Rademacher K.H., Lendvay A. (2014). Preferences for a potential longer-acting injectable contraceptive: Perspectives from women, providers, and policy makers in Kenya and Rwanda. Glob. Health Sci. Pract..

[15] Hard M.L., Mills R.J., Sadler B.M., Wehr A.Y., Weiden P.J., von Moltke L. (2017). Pharmacokinetic Profile of a 2-Month Dose Regimen of Aripiprazole Lauroxil: A Phase I Study and a Population Pharmacokinetic Model. CNS Drugs.

[16] Curley P., Liptrott N.J., Owen A. (2018). Advances in nanomedicine drug delivery applications for HIV therapy. Future Sci. OA.

[17] Verloes R., Klooster G.v., Baert L., Velsen F.v., Bouche M.-P., Spittaels K., Leempoels J., Williams P., Kraus G., Wigerinck P. TMC278 long acting—A parenteral nanosuspension formulation that provides sustained clinically relevant plasma concentrations in HIV-negative volunteers. Proceedings of the 17th International AIDS Conference.

[18] Spreen W.R., Margolis D.A., Pottage J.C. (2013). Long-acting injectable antiretrovirals for HIV treatment and prevention. Curr. Opin. HIV AIDS.

[19] Sillman B., Bade A.N., Dash P.K., Bhargavan B., Kocher T., Mathews S., Su H., Kanmogne G.D., Poluektova L.Y., Gorantla S. (2018). Creation of a long-acting nanoformulated dolutegravir. Nat. Commun..

[20] Kraft J.C., McConnachie L.A., Koehn J., Kinman L., Collins C., Shen D.D., Collier A.C., Ho R.J. (2017). Long-acting combination anti-HIV drug suspension enhances and sustains higher drug levels in lymph node cells than in blood cells and plasma. AIDS.

[21] Tatham L.M., Savage A.C., Dwyer A., Siccardi M., Scott T., Vourvahis M., Clark A., Rannard S.P., Owen A. (2018). Towards a Maraviroc Long-Acting Injectable Nanoformulation. Eur. J. Pharm. Biopharm..

[22] Cory T.J., Midde N.M., Rao P., Kumar S. (2015). Investigational reverse transcriptase inhibitors for the treatment of HIV. Expert Opin. Investig. Drugs.

[23] Hobson J.J., Al-Khouja A., Curley P., Meyers D., Flexner C., Siccardi M., Owen A., Meyers C.F., Rannard S.P. (2019). Semi-solid prodrug nanoparticles for long-acting delivery of water-soluble antiretroviral drugs within combination HIV therapies. Nat. Commun..

[24] Jackson A., Moyle G., Watson V., Tjia J., Ammara A., Back D., Mohabeer M., Gazzard B., Boffito M. (2013). Tenofovir, emtricitabine intracellular and plasma, and efavirenz plasma concentration decay following drug intake cessation: Implications for HIV treatment and prevention. J. Acquir. Immune Defic. Syndr..

[25] R Core Team (2020). R: A Language and Environment for Statistical Computing.

[26] Borchers H.W. (2019). Pracma: Practical Numerical Math Functions. R Package Version 2.2.9. https://CRAN.R-project.org/package=pracma.

[27] Dash P.K., Gendelman H.E., Roy U., Balkundi S., Alnouti Y., Mosley R.L., Gelbard H.A., McMillan J., Gorantla S., Poluektova L.Y. (2012). Long-acting nanoformulated antiretroviral therapy elicits potent antiretroviral and neuroprotective responses in HIV-1-infected humanized mice. AIDS.

[28] Dagur R.S., Branch-Woods A., Mathews S., Joshi P.S., Quadros R.M., Harms D.W., Cheng Y., Miles S.M., Pirruccello S.J., Gurumurthy C.B. (2019). Human-like NSG mouse glycoproteins sialylation pattern changes the phenotype of human lymphocytes and sensitivity to HIV-1 infection. BMC Immunol..

[29] Su H., Sravanam S., Gorantla S., Kaminski R., Khalili K., Poluektova L., Gendelman H.E., Dash P.K. (2020). Amplification of Replication Competent HIV-1 by Adoptive Transfer of Human Cells From Infected Humanized Mice. Front. Cell. Infect. Microbiol..

[30] Yanez J.A., Remsberg C.M., Sayre C.L., Forrest M.L., Davies N.M. (2011). Flip-flop pharmacokinetics—Delivering a reversal of disposition: Challenges and opportunities during drug development. Ther. Deliv..

[31] Nirogi R., Bhyrapuneni G., Kandikere V., Muddana N., Saralaya R., Komarneni P., Mudigonda K., Mukkanti K. (2012). Pharmacokinetic profiling of efavirenz-emtricitabine-tenofovir fixed dose combination in pregnant and non-pregnant rats. Biopharm. Drug Dispos..

[32] Wolfensohn S., Lloyd M. (2008). Handbook of Laboratory Animal Management and Welfare.

[33] Diehl K.H., Hull R., Morton D., Pfister R., Rabemampianina Y., Smith D., Vidal J.M., van de Vorstenbosch C., European Federation of Pharmaceutical Industries Associations, European Centre for the Validation of Alternative Methods (2001). A good practice guide to the administration of substances and removal of blood, including routes and volumes. J. Appl. Toxicol..

[34] Molina J.M., Peytavin G., Perusat S., Lascoux-Combes C., Sereni D., Rozenbaum W., Chene G. (2004). Pharmacokinetics of emtricitabine, didanosine and efavirenz administered once-daily for the treatment of HIV-infected adults (pharmacokinetic substudy of the ANRS 091 trial). HIV Med..

[35] Saag M.S. (2006). Emtricitabine, a new antiretroviral agent with activity against HIV and hepatitis B virus. Clin. Infect. Dis..

[36] Labarthe L., Gele T., Gouget H., Benzemrane M.S., Le Calvez P., Legrand N., Lambotte O., Le Grand R., Bourgeois C., Barrail-Tran A. (2022). Pharmacokinetics and tissue distribution of tenofovir, emtricitabine and dolutegravir in mice. J. Antimicrob. Chemother..

[37] Al-Tabakha M.M. (2010). HPMC capsules: Current status and future prospects. J. Pharm. Pharm. Sci..

[38] Darville N., van Heerden M., Marien D., De Meulder M., Rossenu S., Vermeulen A., Vynckier A., De Jonghe S., Sterkens P., Annaert P. (2016). The effect of macrophage and angiogenesis inhibition on the drug release and absorption from an intramuscular sustained-release paliperidone palmitate suspension. J. Control. Release.

[39] Owen A., Curley P. (2014). Species similarities and differences in pharmacokinetics and distribution of antiretroviral drugs. Humanized Mice for HIV Research.

[40] Rajoli R.K., Back D.J., Rannard S., Freel Meyers C.L., Flexner C., Owen A., Siccardi M. (2015). Physiologically Based Pharmacokinetic Modelling to Inform Development of Intramuscular Long-Acting Nanoformulations for HIV. Clin. Pharm..

