# Supplementary material for: Preclinical Evaluation of Long-Acting Emtricitabine Semi-Solid Prodrug Nanoparticle Formulations
Source: Pharmaceutics. 2023 Jun 27;15(7):1835. doi: 10.3390/pharmaceutics15071835 (PMC10383755; doi:10.3390/pharmaceutics15071835)
Supplement: Supplementary file 1 [file pharmaceutics-15-01835-s001.zip › pharmaceutics-2437549-supplementary.pdf]

		Intra-day			Inter-day		
		Average ± SD (ng/mL)	Variance of accuracy (%)	Variance of precision (%)	Average ± SD (ng/mL)	Variance of accuracy (%)	Variance of precision (%)
Assay 1	5ng/mL	4.5 ±0.23	-10.4	5.1	4.7 ±0.64	-6.3	13.6
	200ng/mL	193.7 ±7.26	-3.2	3.8	191.82 ±11.05	-4.1	5.8
	400ng/mL	388.3 ±9.09	-2.9	2.3	386.8 ±6.23	-3.3	1.6
Assay 2	5ng/mL	4.2 ± 0.19	-16.4	4.6			
	200ng/mL	180.0 ±2.83	-10.2	1.6			
	400ng/mL	380.0 ±7.60	-5.0	2.0			
Assay 3	5ng/mL	5.4 ±0.30	8.0	5.5			
	200ng/mL	201.8 ±4.91	0.9	2.4			
	400ng/mL	392.2 ±12.83	-2.0	3.3			

Table S1 shows the intra-assay variance in accuracy and precision of 3 repetitions of the assay. Also shown is the variance in accuracy and precision of the inter-day assay performance. Accuracy and precision were assessed in triplicate at 3 levels (low (5ng/mL), medium (200ng/mL) and high (400ng/mL)

Figure	MW_Test_group	Challenge	Tissue	Days_Post_Inj	MW_test	p.value	bonf.p.value
4A	1	7	Plasma	14	Untreated_vs_SDN9	0.002	0.006
4A	1	7	Plasma	14	Untreated_vs_SDN10	0.002	0.006
4A	1	7	Plasma	14	SDN10_vs_SDN9	1.000	3.000
4B	2	7	Plasma	28	Untreated_vs_SDN9	0.002	0.006
4B	2	7	Plasma	28	Untreated_vs_SDN10	0.002	0.006
4B	2	7	Plasma	28	SDN10_vs_SDN9	1.000	3.000
5A	3	7	Spleen	28	Untreated_vs_SDN9	0.002	0.006
5A	3	7	Spleen	28	Untreated_vs_SDN10	0.002	0.006
5A	3	7	Spleen	28	SDN10_vs_SDN9	1.000	3.000
5B	4	7	lung	28	Untreated_vs_SDN9	0.002	0.006
5B	4	7	lung	28	Untreated_vs_SDN10	0.002	0.006
5B	4	7	lung	28	SDN10_vs_SDN9	1.000	3.000
5C	5	7	Liver	28	Untreated_vs_SDN9	0.001	0.002
5C	5	7	Liver	28	Untreated_vs_SDN10	0.001	0.002
5C	5	7	Liver	28	SDN10_vs_SDN9	1.000	3.000
4C	6	14	Plasma	14	Untreated_vs_SDN9	0.003	0.008
4C	6	14	Plasma	14	Untreated_vs_SDN10	0.040	0.119
4C	6	14	Plasma	14	SDN10_vs_SDN9	0.070	0.210
4D	7	14	Plasma	28	Untreated_vs_SDN9	0.003	0.008
4D	7	14	Plasma	28	Untreated_vs_SDN10	0.024	0.073
4D	7	14	Plasma	28	SDN10_vs_SDN9	0.027	0.080
5D	8	14	Spleen	28	Untreated_vs_SDN9	0.003	0.008
5D	8	14	Spleen	28	Untreated_vs_SDN10	0.448	1.343

5D	8	14	Spleen	28	SDN10_vs_SDN9	0.027	0.080
5E	9	14	lung	28	Untreated_vs_SDN9	0.003	0.008
5E	9	14	lung	28	Untreated_vs_SDN10	0.552	1.655
5E	9	14	lung	28	SDN10_vs_SDN9	0.009	0.028
5F	10	14	Liver	28	Untreated_vs_SDN9	0.003	0.008
5F	10	14	Liver	28	Untreated_vs_SDN10	0.500	1.500
5F	10	14	Liver	28	SDN10_vs_SDN9	0.009	0.028

Table S2 Differences in viral load in plasma and tissues at timepoints 14 and 28 days following challenge 7 or 14 days after dosing of SDN9, SDN10 or control/no treatment, were tested for significance using a one-sided, pairwise Mann-Whitney-Wilcoxon test, with Bonferroni correction factor of 3 (for 3 pairwise comparison tests per challenge/tissue/timepoint group).
